# Novel Insights into the Mode of Action of Vasorelaxant Synthetic Polyoxygenated Chalcones

**DOI:** 10.3390/ijms21051609

**Published:** 2020-02-26

**Authors:** Samuel Legeay, Kien Trân, Yannick Abatuci, Sébastien Faure, Jean-Jacques Helesbeux

**Affiliations:** 1 MINT, UNIV Angers, INSERM 1066, CNRS 6021, IRIS-IBS-CHU, 4 Rue Larrey, 49100 Angers, France; sebastien.faure@univ-angers.fr; 2 SONAS, EA921, UNIV Angers, SFR QUASAV, Faculty of Health Sciences, Dpt Pharmacy, 16 Bd Daviers, 49045 Angers CEDEX 01, France; Kien.Tran@usherbrooke.ca (K.T.); yannick.abatuci@univ-angers.fr (Y.A.); jean-jacques.helesbeux@univ-angers.fr (J.-J.H.)

**Keywords:** polyphenols, chalcones, structure-activity relationships, endothelium, vasodilation, cardiovascular diseases

## Abstract

Polyphenols consumption has been associated with a lower risk of cardiovascular diseases (CVDs) notably through nitric oxide (NO)- and estrogen receptor α (ERα)-dependent pathways. Among polyphenolic compounds, chalcones have been suggested to prevent endothelial dysfunction and hypertension. However, the involvement of both the NO and the ERα pathways for the beneficial vascular effects of chalcones has never been demonstrated. In this study, we aimed to identify chalcones with high vasorelaxation potential and to characterize the signaling pathways in relation to ERα signaling and NO involvement. The evaluation of vasorelaxation potential was performed by myography on wild-type (WT) and ERα knock-out (ERα-KO) mice aorta in the presence or in absence of the eNOS inhibitor Nω-nitro-L-arginine methyl ester (L-NAME). Among the set of chalcones that were synthesized, four (**3**, **8**, **13** and **15**) exhibited a strong vasorelaxant effect (more than 80% vasorelaxation) while five compounds (**6, 10, 11, 16, 17**) have shown a 60% relief of the pre-contraction and four compounds (**12**, **14**, **18**, **20**) led to a lower vasorelaxation. We were able to demonstrate that the vasorelaxant effect of two highly active chalcones was either ERα-dependent and NO-independent or ERα-independent and NO-dependent. Thus some structure-activity relationships (SAR) were discussed for an optimized vasorelaxant effect.

## 1. Introduction

According to the World Health Organization (WHO), cardiovascular diseases are the first leading cause of death worldwide [1]. Most cardiovascular diseases (CVDs) are initially due to hypertension and/or atherosclerosis that could be initiated by endothelial dysfunction [2,3]. These pathological conditions lead to alterations of the endothelium resulting in imbalanced endothelial factors, including a decrease in nitric oxide (NO) bioavailability, vascular constriction, and platelet activation.

Interestingly, epidemiological studies indicate that the prevalence of CVDs is lower in premenopausal women compared to age-matched men and significantly increases in women after menopause, suggesting a protective role of estrogens in premenopausal women [4,5]. Both estrogen receptor α (ERα) and estrogen receptor β (ERβ) are involved in cardiovascular protection with a predominant role of ERα at the vascular level [6,7]. ERα, expressed by endothelial cells and co-localized with endothelial NO synthase (eNOS), has been identified as an eNOS activator through an Src/ERK1/2-dependent pathway both in human primary endothelial cells and mice [8,9]. These endothelial effects are strengthened by clinical data reporting that menopausal hormone replacement therapy (MHT) prevents cardiovascular outcomes in perimenopausal women, confirming the role of estrogen receptor activation for the prevention of the endothelial dysfunction in women [10,11].

Various studies have suggested that polyphenolic compounds consumption may prevent cardiovascular diseases [12,13,14]. Among polyphenols, chalcones, which belong to the flavonoid family and are ubiquitously found in fruits, vegetables, wine, and tea, have been described to favor vascular functions [15]. Recently, the chalcone isoliquiritigenin has been identified to induce an endothelium-independent vasodilation through a calcium-activated potassium channel-dependent (BK_Ca_) pathway in mice [15]. These results are not consistent with those obtained with dihydrospinochalcone-A which induced an endothelium-dependent vasodilation through a NO/sGC/PKG pathway [16]. Meanwhile, chalcones have been identified to exert an estrogenic activity notably through modulation of ERα [17]. Moreover, flavonoids-induced vasodilation through an ERα dependent pathway has already been observed both with rats and mice aortas [8,9,18,19]. However, the precise involvement of ERα in the chalcones-induced vasodilation has not been studied so far.

The aim of this work was (i) to synthesize and characterize polyoxygenated chalcones, (ii) to evaluate their vasorelaxant potential on mice thoracic aorta, (iii) to assess the involvement of ERα and NO signaling pathways and (iv), to identify structure-activity relationships (SAR) for a high vasorelaxant efficacy and potency in order to complete the database of vasodilators which can be used for the prevention and/or the treatment of vascular diseases.

## 2. Results

### 2.1. Chemistry and cLogP

**Scheme 1:**
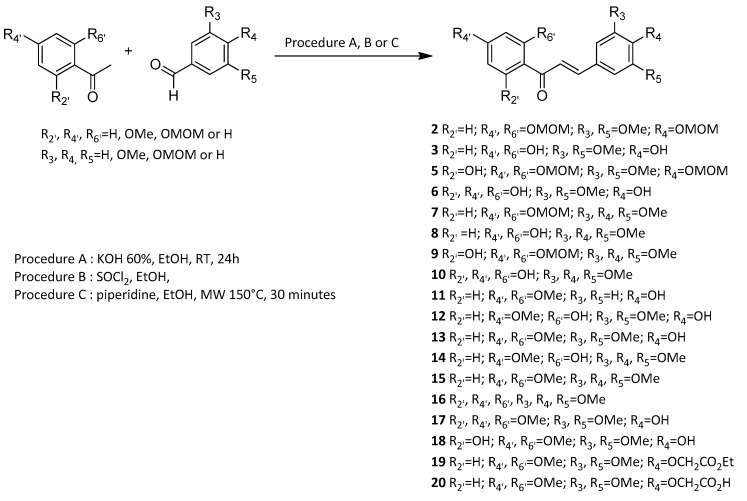
General scheme for the synthesis of chalcones. Synthetic chalcones were synthesized through the coupling of the corresponding either MOM-protected or phenolic acetophenones and benzaldehydes. Hydrolysis of MOM groups or selective demethylation in the presence of AlCl_3_ were achieved. These procedures led to thirteen synthetic polyoxygenated chalcones whose vasorelaxant effect was evaluated.

Synthetic polyhydroxylated chalcones **3**, **6**, **8** and **10** were synthesized through a two-step procedure starting from the corresponding methoxymethyl (MOM)-protected acetophenones and benzaldehydes. Although this strategy required an extra deprotection step compared to Claisen-Schmidt condensation of phenolic starting materials, it has led to a better overall yield as observed for the synthesis of chalcone **8** compared to literature data [20,21]. Due to reactivity and efficiency issues in basic medium, chalcones **11**, **13** and **17** bearing a hydroxyl (OH) group in the C-4 position have been prepared through an acid-catalyzed coupling mechanism using thionyl chloride (SOCl_2_) in ethanol (EtOH). Thus it avoided the need for a synthesis strategy with a MOM protection-deprotection approach [22]. Nevertheless, in situ generated hydrochloric acid (HCl) catalysis was unsuccessful when applied to the synthesis of diphenolic chalcone **18**. Thus, it has been prepared under microwave (MW) irradiations in the presence of piperidine [23]. In this work two other synthetic chalcones **12** and **14**, bearing a phenol function on the C-2_’_ position, have been prepared through selective demethylation of the corresponding 2’-methoxychalcones **13** and **15** in the presence of aluminum chloride (AlCl_3_) [24]. It is worth mentioning that the demethylation of hexamethoxychalcone **16** led only to **17** (52% yield) without any trace of **18** (Scheme 1). Finally, the calculated octanol-water partition coefficient (cLogP) were similar, ranging from 1.80 for synthetic chalcone **6** to 3.33 for synthetic chalcone **14** (Table 1).

### 2.2. Evaluation of Vasorelaxant Activity

**Figure 1:**
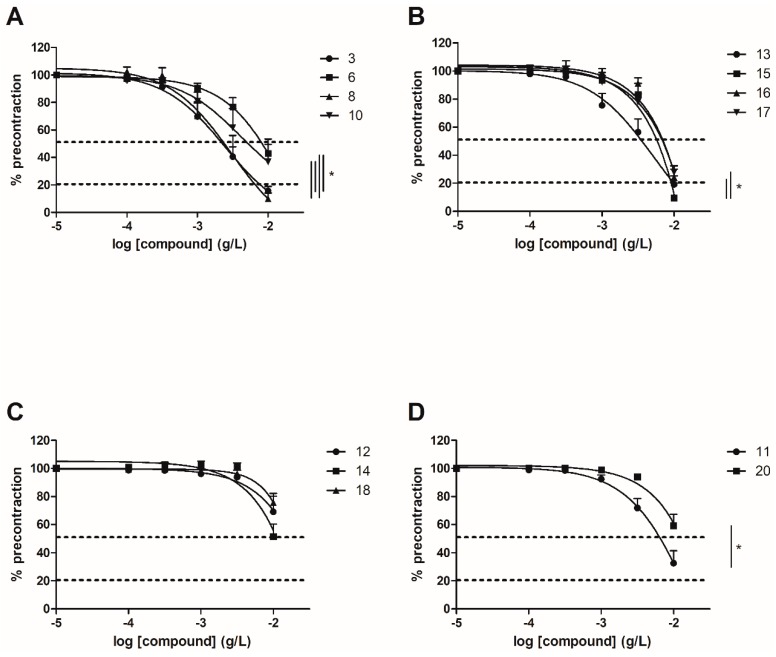
Concentration-response curves for the vasorelaxation effect on WT mice thoracic aorta of synthetic polyoxygenated chalcones. **A**: vasorelaxation effect of chalcones with R_2’_ = R_4’_ = OH and R_6’_ = H or OH; **B**: vasorelaxation effect of chalcones with R_2’_ = R_4’_ = OCH_3_ and R_6’_ = H or OCH_3_; **C**: vasorelaxation effect of chalcones with R_2’_ = OH, R_4’_ = OCH_3_ and R_6’_ = H or OCH_3_; **D**: vasorelaxation effect of chalcones with R_2’_ = R_4’_ = OCH_3_ and R_6’_ = H. Mice were ovariectomized 7 days before the experiment. Vessels were pre-contracted with U46619, phenylephrine, and serotonin, at 80 % of their maximal response. The presence of functional endothelium was assessed by determining the ability of acetylcholine (10 µM) to induce more than 50% relaxation of pre-contracted rings. Results are expressed in percentage of precontraction (*y*-axis) following the concentration (*x*-axis). Compounds **3**, **8**, **13** and **15** induced higher vasorelaxation, over 80%. Compounds **6**, **10**, **11**, **16** and **17** induced an average vasorelaxation between 50% to 80%. Compounds **12**, **14**, **18** and **20** exerted a low vasorelaxation effect below 50%. *N* = 4–7, * *p* < 0.05 (Mann-Whitney).

Vasorelaxation induced by each synthetic chalcone was first evaluated on WT mice thoracic aorta rings. Compounds **3**, **8**, **13** and **15** with identical substituents at C-2’ and C-4’ positions, absence of substituent at the C-6’ position and presence of OH and/or OCH_3_ groups at C-3, C-4 and C-5 positions induced a high vasorelaxation reaching more than 80%. Compounds **6**, **10**, **11**, **16** and **17** with OH or OCH_3_ group at the C-6’ position or unsubstituted at C-3 and C-5 positions induced an average vasorelaxation between 50% to 80%. Finally, compounds **12**, **14**, **18** and **20** with both OH and OCH_3_ groups at C-2’, C-4’ and/or C-6’ positions or a bulkier OCH_2_COOH group at C-3 position exerted a low vasorelaxation effect below 50% (Figure 1 and Table 2).

### 2.3. Involvement of ERα and NO Pathways

#### 2.3.1. Evaluation of Vasorelaxant Activity on ERα KO Mice Aorta

**Figure 2:**
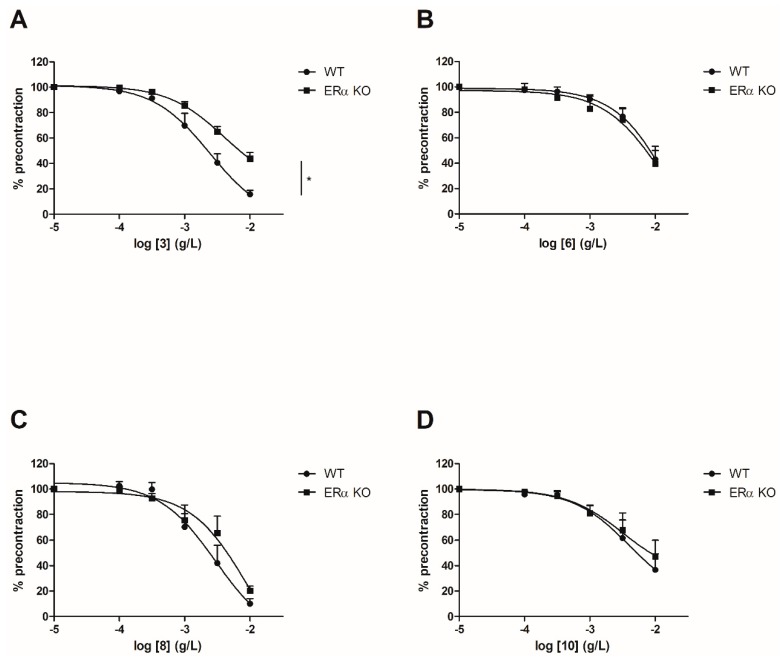
Concentration-response curves for the vasorelaxation effect on both WT and ERαKO mice thoracic aorta of synthetic chalcones **3** (**A**), **6** (**B**), **8** (**C**) and **10** (**D**). Mice were ovariectomized 7 days before the experiment. Vessels were pre-contracted with U46619, phenylephrine, and serotonin, at 80 % of their maximal response. The presence of functional endothelium was assessed by determining the ability of acetylcholine (10 µM) to induce more than 50% relaxation of pre-contracted rings. Results are expressed in percentage of precontraction (*y*-axis) following the concentration (*x*-axis). Only the synthetic chalcone **3** induced a significant lower vasorelaxation in ERα KO mice thoracic aorta rings compared to WT mice thoracic aorta rings (56.23 ± 4.91% vs. 84.28 ± 3.24% respectively) while no significant differences were observed with chalcones **6**, **8** and **10**. *N* = 4–7, * *p* < 0.05 (Mann-Whitney).

**Figure 3:**
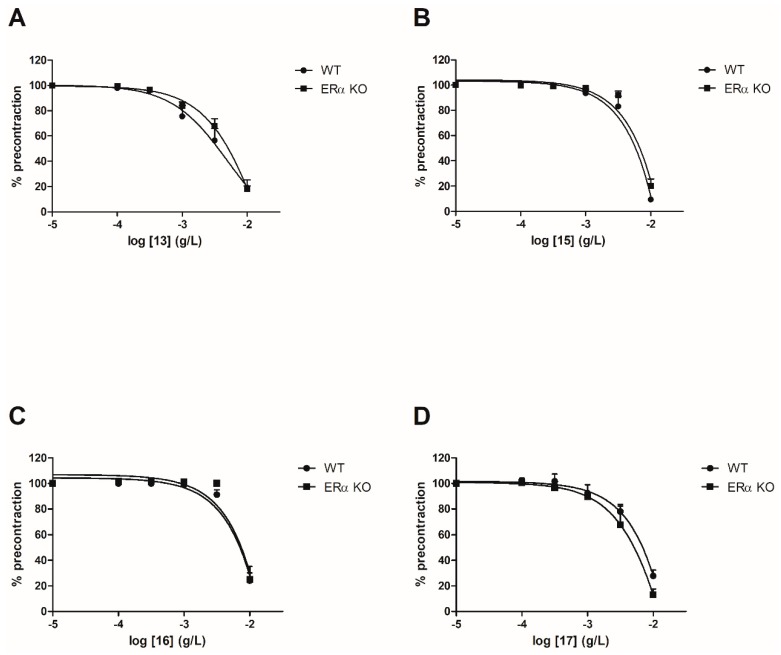
Concentration-response curves for the vasorelaxation effect on both WT and ERαKO mice thoracic aorta of synthetic chalcones **13** (**A**), **15** (**B**), **16** (**C**) and **17** (**D**). Mice were ovariectomized 7 days before the experiment. Vessels were pre-contracted with U46619, phenylephrine, and serotonin, at 80 % of their maximal response. The presence of functional endothelium was assessed by determining the ability of acetylcholine (10 µM) to induce more than 50% relaxation of pre-contracted rings. Results are expressed in percentage of precontraction (*y*-axis) following the concentration (*x*-axis). No significant differences were observed with synthetic chalcones **13**, **15**, **16** and **17**. *N* = 4–7 (Mann-Whitney).

To evaluate the involvement of the ERα pathway in the chalcones-induced vasorelaxation, experiments have been conducted on ERα KO mice aortas. To focus on the role of OH or OCH_3_ groups at C-6’ position with synthetic chalcones exhibiting a high vasorelaxant effect, compounds **3**, **6**, **8**, **10**, **13**, **15**, **16** and **17** have been tested on ERα KO mice thoracic aorta rings. Interestingly, only the synthetic chalcone **3** induced a significant lower vasorelaxation in ERα KO mice thoracic aorta rings compared to WT mice thoracic aorta rings (56.23 ± 4.91% vs. 84.28 ± 3.24%, respectively) while no significant differences were observed with synthetic chalcones **6**, **8**, **10**, **13**, **15**, **16** and **17** (Figure 2 and Figure 3 and Table 2).

#### 2.3.2. Evaluation of Vasorelaxant Activity in the Presence of ***Nω-nitro-L-arginine methyl ester*** (L-NAME)

**Figure 4:**
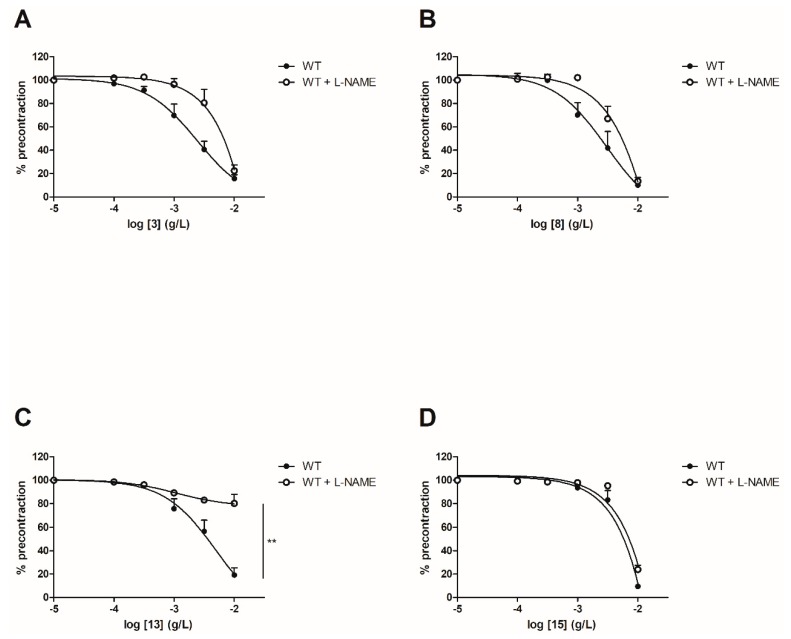
Concentration-response curves for the vasorelaxation effect on WT mice thoracic aorta of synthetic chalcones **3** (**A**), **8** (**B**), **13** (**C**) and **15** (**D**) in the presence or in absence of L-NAME, a selective eNOS inhibitor. Mice were ovariectomized 7 days before the experiment. Vessels were pre-contracted with U46619, phenylephrine, and serotonin, at 80 % of their maximal response. The presence of functional endothelium was assessed by determining the ability of acetylcholine (10 µM) to induce more than 50% relaxation of pre-contracted rings. Results are expressed in percentage of precontraction (*y*-axis) following the concentration (*x*-axis). L-NAME only prevented the chalcone-induced vasorelaxation for the synthetic chalcone **13** at the highest concentration (10^−2^ g/L) and seemed to prevent the chalcone-induced vasorelaxation for compounds **3** and **8** at low concentrations. No difference was observed for chalcone **15**. *N* = 5–7, ** *p* < 0.01 (Mann-Whitney).

**Figure 5:**
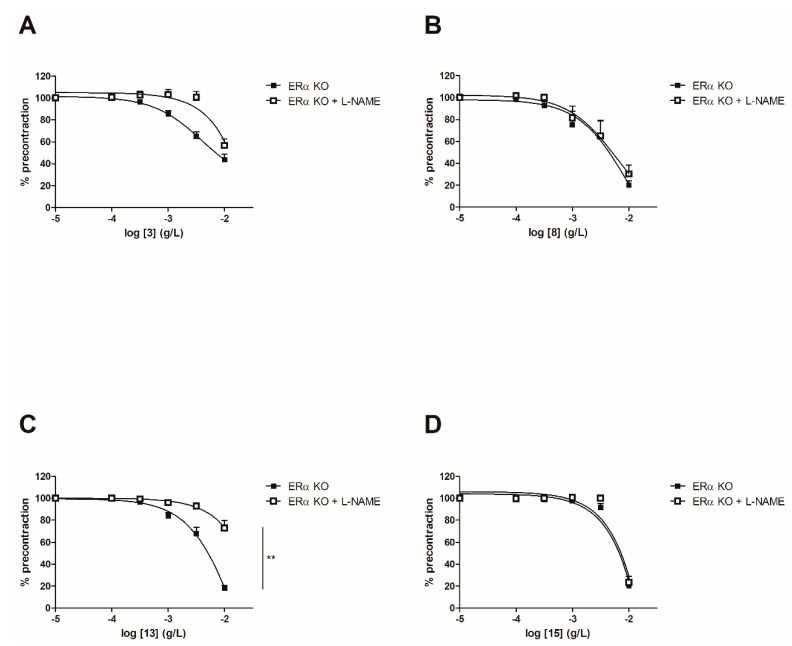
Concentration-response curves for the vasorelaxation effect on ERαKO mice thoracic aorta of synthetic chalcones **3** (**A**), **8** (**B**), **13** (**C**) and **15** (**D**). Mice were ovariectomized 7 days before the experiment. Vessels were pre-contracted with U46619, phenylephrine, and serotonin, at 80 % of their maximal response. The presence of functional endothelium was assessed by determining the ability of acetylcholine (10 µM) to induce more than 50% relaxation of pre-contracted rings. Results are expressed in percentage of precontraction (*y*-axis) following the concentration (*x*-axis). L-NAME only prevented the chalcone **13**-induced vasorelaxation (81.96 ± 2.46% vs. 26.96 ± 6.72%) while having no effect with synthetic chalcones **3**, **8** and **15**. *N* = 4–7, ** *p* < 0.01 (Mann-Whitney).

In order to evaluate the involvement of the NO pathway, experiments have then been performed after incubation for 20 min with NOS inhibitor (10^−4^ M) on both WT and ERα KO mice thoracic aorta rings. To focus on the role of groups at C-2’, C-4’ and C-4 positions, synthetic chalcones **3**, **8**, **13** and **15** have been tested. Interestingly, L-NAME prevented the chalcone-induced vasorelaxation only for chalcone **13** in WT mice thoracic aorta rings at the highest concentration (10^−2^ g/L, Figure 4 and Table 2). It is noteworthy that L-NAME seemed to prevent the chalcone-induced vasorelaxation for compounds **3** and **8** at low concentrations and this effect is not recovered at 10^−2^ g/L. No difference was observed for the synthetic chalcone **15** (Figure 4A,B). Similarly, the presence of L-NAME on ERα KO mice thoracic aorta rings prevented the vasorelaxation only with the synthetic chalcone **13** (81.96 ± 2.46% vs. 26.96 ± 6.72%) while having no effect with synthetic chalcones **3**, **8** and **15** (Figure 5 and Table 2).

## 3. Discussion

By inhibiting the angiotensin-converting enzyme (ACE) and inducing high vasodilation, the anti-hypertensive effect of amino-chalcones has been demonstrated in vitro and in vivo on rats, rabbits, and dogs [25,26]. Several molecular targets such as cyclooxygenase [27] and potassium channels [28], metabolism pathways such as triacylglycerol synthesis pathway [29,30,31] and pro-angiogenic properties [32] have also been identified to explain the potential role of chalcones in the prevention and/or treatment of cardiovascular diseases. However, all these effects have been mainly highlighted with functionalized chalcones bearing amino, prenyl or sulfonyloxy groups and little data are available for polyoxygenated chalcones [25].

The present study described the synthesis of thirteen synthetic chalcones bearing oxygenated substituents (OH and OCH_3_). The pharmacological characterization of their vascular activity including the involvement of NO and ERα-dependent pathways allowed the identification of two promising compounds (**3** and **13**) inducing an endothelium-dependent vasorelaxation. Precisely, the synthetic chalcone **3** is preferable for an ERα-dependent vasorelaxation while the synthetic chalcone **13** is preferable for an ERα-independent and NO-dependent vasorelaxation.

ERα has already been identified as a target for chalcones, especially in the field of cancer [17,33] and it is now well accepted that estrogens can protect against cardiovascular diseases [11,34,35]. At the vascular level, ERα pathway can, through a non-genomic mechanism, lead to an endothelium-dependent vasodilation [34,36]. The identification in this work of compounds **3** and **13** with an ERα- or NO-dependent mechanism, respectively, clearly confirms that these two chalcones exert an endothelium-dependent vasorelaxation in mice thoracic aorta.

Other vascular mechanisms as phosphodiesterase (PDE) inhibition or calcium channel blocking have also been described to disclose chalcones-induced vasorelaxation [9,15]. These targets, mainly located on vascular smooth muscle cells, would explain the NO- and ERα-independent vasorelaxation of the other synthetic chalcones tested. In addition, these mechanisms may also be involved to explain the remaining vasorelaxation observed on ERαKO mice in the presence of L-NAME. Further studies are however required to confirm the involvement of PDE or calcium channel in the NO- and ERα-independent vasorelaxation of the present synthetic chalcones.

Modifications of the substitution pattern contributed to the study of structure-activity relationships. The synthetic chalcone **14**, bearing an OH group at C-2’ position and an OCH_3_ group at C-4’ position exerted a low vasorelaxation effect (less than 50%). Synthetic chalcones **6**, **10**, **16**, **17** and **18**, bearing an OH group or an OCH_3_ group at the C-6’ position, exerted an average or a low vasorelaxation effect (less than 80%). Altogether, the data collected here demonstrated that both C-2’ and C-4’ positions must bear the same group, either OH or OCH_3_, and an unsubstituted C-6’ position is required for a better activity. Accordingly, in a study evaluating the relaxant potency and efficacy in rat thoracic aorta of prenylated and allylated chalcones, an increased vasorelaxant efficacy was also identified for chalcones without any OH group at the C-6’ position [37]. Additionally, the number of OH groups bearing by the chalcone backbone was also described as an important factor for a higher activity [37]. The replacement of OH groups by OCH_3_ groups revealed that the corresponding compounds did not exhibit a higher relaxant efficacy as demonstrated with compound **3** and **8** compared to compound **13** and **15**. These data confirmed the importance of the number of OH groups but they also highlighted that OCH_3_ groups must be considered. More precisely, we identified that the 3,5-dimethoxy-4-hydroxybenzene moiety is required for both an ERα- or NO-dependent vasorelaxation in both 2’,4’-dihydroxychalcone or 2’,4’-dimethoxychalcone series.

Recently, isoliquiritigenin has been described to induce an endothelium-independent vasodilation through a calcium-activated potassium channel-dependent (BK_Ca_) pathway in mice [15]. By substituting OH groups by OCH_3_ groups at the C-2’ and C-4’ positions, isoliquiritigenin derivatives such as compound **11** have been synthsized. This synthetic chalcone exerted a comparable vasorelaxation than isoliquiritigenin through an endothelium-independent pathway. The involvement of the BK_Ca_ in compound **11**-induced vasorelaxation now needs to be explored in order to further understand the role of the methoxylation at C-2’ and C-4’ positions in the BK_Ca_-dependent vasorelaxation.

Similar cLogP, ranging from 1.80 to 3.33 were observed for the thirteen synthesized chalcones. We and others demonstrated that a higher cLogP value of flavonoids was associated with a higher vasorelaxation potential [9,38]. However, this association has been observed for flavonoids with very low lipophilicity (cLogP < 1) as anthocyanidins [9]. In the present study, no association between cLogP and the vasorelaxant potential has been observed, likely because of cLogP mainly ranging around 2 and 3.

In conclusion, coupling reactions between acetophenones and benzaldehydes led to thirteen synthetic polyoxygenated chalcones bearing OH groups and/or OCH_3_ groups and/or a bulkier OCH_2_COOH group. The vascular effects of chalcones were assessed on WT and ERα KO mice thoracic aorta and revealed four compounds with a very high vasorelaxation potential (**3**, **8**, **13** and **15**). More precisely, the synthetic chalcone **3** exerted an ERα-dependent and NO-independent vasorelaxation while the synthetic chalcone **13** exerted an ERα-independent and NO-dependent vasorelaxation. Structure-activity relationships (SAR) study allowed us to identify the 3,5-dimethoxy-4-hydroxybenzene moiety as a pharmacophore for both an ERα- or NO-dependent vasorelaxation. Finally, the current study provides new promising data for the preventive and therapeutic use of chalcones in the field of CVDs.

## 4. Materials and Methods

### 4.1. Chemistry

All chemicals were purchased from Acros Organics (Geel, Belgium). Chromatographic separations for purification processes were achieved with flash chromatography IntelliFlash 310 (Analogix) using pre-packed C18 (Interchim) or silica gel column Chromabond® flash RS column (Macherey-Nagel). ^1^H and ^13^C NMR were recorded on Jeol GSX WB 270MHz. Chemical shifts are reported in parts per million (δ ppm).

**Scheme 2:**
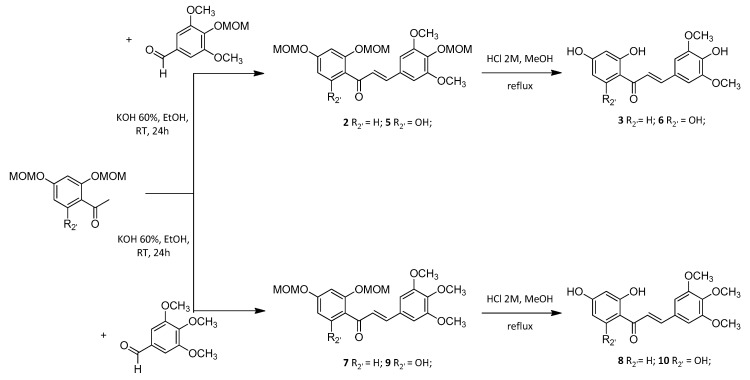
Strategy for Procedure A.

Different procedures (A, B or C) have been used to achieve the Claisen Schmidt condensation (Scheme 2).

Procedure A: To a solution of acetophenone (0.5 mmol) in ethanol are added benzaldehyde (0.5 mmol) and a 60% solution of potassium hydroxide (KOH). The resulting reaction mixture is stirred at room temperature for 24 H. Then 10 mL of water is added. The corresponding chalcone is collected by vacuum filtration or purified by silica gel flash chromatography. When MOM protecting groups are used, their hydrolysis is undertaken with 2N HCl (2 eq/MOM group) at reflux in a methanol solution. Completion of the reaction is determined by thin-layer chromatography (TLC). After cooling at room temperature (RT), water is added to the reaction mixture and the chalcone is extracted three times with ethyl acetate (EtOAc). The final product is purified by column chromatography.

*(E)-1-(2,4-dimethoxymethylphenyl)-3-(4-methoxymethyl-3,5-dimethoxyphenyl)prop-2-en-1-one* (**2**). Procedure A was applied starting from 226 mg of 4-methoxymethyl-3,5-dimethoxybenzaldehyde (1 mmol) and 240 mg (1 mmol) of 2,4-dimethoxymethylacetophenone. Yield: 63%.^1^H NMR (270 MHz, CDCl_3_): δ 7.64 (d, *J* = 8.6 Hz, 1H), 7.54 (d, *J* = 15.7 Hz, 1H), 7.34 (d, *J* = 15.7 Hz, 1H), 6.82 (m, 3H), 6.76 (d, *J* = 8.6 Hz, 1H), 5.24 (s, 2H), 5.21 (s, 2H), 5.16 (s, 2H), 3.87 (s, 3H), 3.60 (s, 3H), 3.49 (s, 3H).^13^C NMR (68 MHz, CDCl_3_): δ 191.1, 161.3, 157.4, 153.6, 142.7, 136.5, 132.1, 131.2, 126.6, 124.0, 109.2, 105.4, 103.5, 98.2, 95.1, 94.2, 57.1, 56.5, 56.2, 56.0.

*(E)-1-(2,4-dihydroxyphenyl)-3-(4-hydroxy-3,5-dimethoxyphenyl)prop-2-en-1-one* (**3**). Hydrolysis was performed starting from **2** (83 mg, 0.18 mmol) leading to 18 mg of **3**. Yield: 30%. ^1^H NMR (270 MHz, CD_3_OD): δ 8.06 (d, *J* = 9.0 Hz, 1H), 7.78 (d, *J* = 15.2 Hz, 1H), 7.68 (d, *J* = 15.4 Hz, 1H), 7.08 (s, 2H), 6.41 (dd, *J* = 8.9, 2.4 Hz, 1H), 6.29 (d, *J* = 2.4 Hz, 1H), 3.93 (s, 3H).^13^C NMR (68 MHz, CD_3_OD): δ 194.6, 168.6, 167.4, 150.6, 147.2, 141.0, 134.6, 128.4, 120.1, 115.8, 110.1, 108.6, 104.7, 57.9.

*(E)-1-(2-hydroxy-4,6-dimethoxymethylphenyl)-3-(4-methoxymethyl-3,5-dimethoxyphenyl)prop-2-en-1-one* (**5**). Procedure A was applied starting from 256 mg of 2-hydroxy-4,6-dimethoxymethylacetophenone (1 mmol) and 226 mg of 4-methoxymethyl-3,5-dimethoxybenzaldehyde (1 mmol). Yield: 92%. ^1^H NMR (270 MHz, CDCl_3_): δ 7.86 (d, *J* = 15.5 Hz, 1H), 7.71 (d, *J* = 15.5 Hz, 1H), 6.85 (s, 2H), 6.32 (d, *J* = 2.3 Hz, 1H), 6.21 (d, *J* = 2.3 Hz, 1H), 5.28 (s, 2H), 5.20 (s, 2H), 5.18 (s, 6H), 3.62 (s), 3.54 (s), 3.49 (s).^13^C NMR (68 MHz, CDCl_3_): δ 192.8, 167.4, 163.6, 159.9, 153.7, 142.6, 131.5, 126.9, 107.6, 105.5, 98.3, 97.6, 95.4, 94.7, 94.0, 57.2, 57.0, 56.5, 56.0.

*(E)-1-(2,4,6-trihydroxyphenyl)-3-(4-hydroxy-3,5-dimethoxyphenyl)prop-2-en-1-one* (**6**). Hydrolysis was performed starting from **5** (141 mg, 0.30 mmol) leading to 28 mg of **6**. Yield: 28%.^1^H NMR (270 MHz, δ 8.08 (d, *J* = 15.5 Hz, 1H), 7.67 (d, *J* = 15.5 Hz, 1H), 6.93 (s, 2H), 5.84 (s, 2H), 3.89 (s, 6H). ^13^C NMR (68 MHz, CD_3_OD): δ 195.0, 167.2, 166.9, 150.6, 144.9, 140.5, 129.0, 127.3, 108.0, 106.9, 97.0, 57.7.

(*E)-1-(2,4-dimethoxymethylphenyl)-3-(3,4,5-trimethoxyphenyl)prop-2-en-1-one* (**7**). Procedure A was applied, starting from 196 mg (1 mmol) of 3,4,5-trimethoxybenzaldehyde and 240 mg (1 mmol) of 2,4-dimethoxymethylacetophenone. Yield: 49%.

*(E)-1-(2,4-dihydroxyphenyl)-3-(3,4,5-trimethoxyphenyl)prop-2-en-1-one* (**8**). Hydrolysis was performed starting from **7** (100 mg, 0.24 mmol) leading to 77 mg of **8**. Yield: 97%. ^1^H NMR (270 MHz, CD_3_OD): δ 8.07 (d, *J* = 9.0 Hz, 1H), 7.77 (s, 2H), 7.09 (s, 2H), 6.43 (dd, *J* = 8.8, 2.4 Hz, 1H), 6.30 (d, *J* = 2.4 Hz, 1H), 3.92 (s, 6H), 3.82 (s, 3H).

*(E)-1-(2-hydroxy-4,6-dimethoxymethylphenyl)-3-(3,4,5-trimethoxyphenyl)prop-2-en-1-one* (**9**). Procedure A was applied starting from 105 mg of 3,4,5-trimethoxybenzaldehyde(0.53 mmol) and 137 mg of 2-hydroxy-4,6-dimethoxymethylacetophenone(0.53 mmol). Yield: 62%.

*(E)-1-(2,4,6-trihydroxyphenyl)-3-(3,4,5-trimethoxyphenyl)prop-2-en-1-one* (**10**). Hydrolysis was performed starting from **9** (80 mg, 0.18 mmol) leading to 59 mg of **10**. Yield: 92%. ^1^H NMR (270 MHz, CD_3_OD): δ 8.13 (d, *J* = 15.5 Hz, 1H), 7.66 (d, *J* = 15.6 Hz, 1H), 6.94 (s, 2H), 5.85 (s, 2H), 3.89 (s, 6H), 3.81 (s, 3H). ^13^C NMR (68 MHz, CD_3_OD) δ 195.0, 167.5, 167.0, 155.8, 143.9, 142.1, 134.0, 129.5, 107.8, 107.0, 97.0, 62.1, 57.6.

*(E)-1-(2,4-dimethoxyphenyl)-3-(3,4,5-trimethoxyphenyl)prop-2-en-1-one* (**15**). Procedure A has been applied using 200 mg (1.11 mmol) of 2,4-dimethoxyacetophenone and 220 mg (1.11 mmol) of 3,4,5-trimethoxybenzaldehyde leading to 275 mg of **15** as a yellow powder (69% yield). ^1^H NMR (CDCl_3_, 270 MHz): δ 7.73 (d, 1H, *J* = 8.6 Hz), 7.57 (d, 1H, *J* = 15.7 Hz), 7.37 (d, 1H, *J* = 15.7 Hz), 6.82 (s, 2H), 6.57 (dd, 1H, *J* = 8.4 Hz, *J* = 2.2 Hz), 6.51 (d, 1H, *J* = 2.4 Hz). ^13^C NMR (CDCl_3_, 68 MHz): δ 194.8, 162.6, 159.1, 151.5, 149.5, 144.9, 128.3, 127.6, 123.2, 112.2, 111.3, 110.3, 91.1, 56.2, 56.2, 56.1, 55.7.HRMS FAB [M + H] 359.1483.

*(E)-1-(2,4,6-trimethoxyphenyl)-3-(3,4,5-trimethoxyphenyl)prop-2-en-1-one* (**16**). Procedure A has been applied using 100 mg of 2,4,6-trimethoxyacetophenone and 93 mg of 3,4,5-trimethoxybenzaldehyde leading to 125 mg of **16** as a light yellow powder (72% yield). ^1^H NMR (CDCl_3_, 270 MHz): δ 7.24 (d, 1H, *J* = 15.9 H), 6.86 (d, 1H, *J* = 15.9 Hz), 6.75 (s, 2H), 6.17 (s, 2H), 3.87 (s, 9H), 3.86 (s, 3H), 3,77 (s, 6H). ^13^C NMR (CDCl_3_, 68 MHz): δ 194.6, 162.6, 159.0, 153.6, 144.6, 140.3, 130.7, 128.8, 112.0, 105.7, 90.9, 61.1, 56.3, 56.1, 55.6. HRMS FAB [M + H] 389.1589.

Procedure B: Thionyl chloride (0.5 eq) is added dropwise to a solution of acetophenone (1 eq) and benzaldehyde (1 eq) in 3 mL of dichloromethane (DCM). After being stirred at room temperature for 24 h, the reaction mixture is quenched with 10 mL of water. Crude chalcone is collected through filtration and could be purified by silica gel column chromatography when it is required. 

*(E)-3-(4-hydroxyphenyl)-1-(2,4-dimethoxyphenyl)prop-2-en-1-one* (**11**). Procedure B was applied starting from 149 mg of 2,4-dimethoxyacetophenone and 101 mg of 4-hydroxybenzaldehyde, leading to 184 mg of **11** as a greenish powder. Yield: 78%. ^1^H NMR (CDCl_3_, 270 MHz): δ 7.73 (d, *J* = 8.5 Hz, 1H), 7.64 (d, 1H, *J* = 15.6 Hz, oléfine), 7.48 (d, 2H, *J* = 8.5 Hz, Ar), 7.37 (d, 1H, *J* = 15.6 Hz, oléfine), 6.88 (d, 2H, *J* = 8.5 Hz, Ar), 6.78 (s, 1H, OH), 6.56 (dd, 1H, *J* = 8.7 Hz, 2.0 Hz, Ar ), 6.49 (d, 1H, *J* = 2.1 Hz, Ar), 3.89 (s, 3H, -OMe), 3.87 (s,3H,-OMe). 13C RMN (CDCl3, 68 MHz): δ 191.7, 164.5, 160.7, 158.5, 143.2, 133.1, 130.6, 128.1, 124.9, 122.6, 116.3, 105.5, 105.4, 99.0, 56.0, 55.8.

*(E)-3-(4-hydroxy-3,5-dimethoxyphenyl)-1-(2,4-dimethoxyphenyl)prop-2-en-1-one* (**13**). Procedure B was applied starting from 250 mg of 2,4-dimethoxyacetophenone and 250 mg of 4-hydroxy-3,5-dimethoxybenzaldehyde, leading, after column chromatography (cyclohexane—EtOAc (6:4)) to 394 mg of **13** as a yellow oil. Yield: 85%. ^1^H NMR (CDCl_3_, 270 MHz): δ 7.71 (d, *J* = 8.5 Hz, 1H), 7.57 (d, *J* = 15.7 Hz, 1H), 7.32 (d, *J* = 15.6 Hz, 1H), 6.57 (dd, *J* = 8.6 Hz, 2.3 Hz, 1H), 6.50 (d, *J* = 2.2 Hz, 1H), 5.79 (s, 1H), 3.93 (s, 6H), 3.90 (s, 3H), 3.88 (s, 3H). ^13^C RMN (CDCl_3_, 68 MHz): δ 191.0, 164.2, 160.4, 147.4, 143.1, 137.2, 132.8, 127.1, 125.6, 122.6, 105.5, 105.3, 98.9, 56.5, 55.9, 55.7.

*(E)-3-(4-hydroxy-3,5-dimethoxyphenyl)-1-(2,4,6-trimethoxyphenyl)prop-2-en-1-one* (**17**). Procedure B was applied starting from 150 mg of 2,4,6-trimethoxyacetophenone and 129 mg of 4-hydroxy-3,5-dimethoxybenzaldehyde, leading to 70 mg of **17** as a yellow powder. Yield: 26%. ^1^H NMR (CDCl_3_, 270 MHz): δ 7.23 (d, *J* = 15.7 Hz, 1H), 6.83 (d, *J* = 15.9 Hz, 1H), 6.76 (s, 2H, Ar), 6.17 (s, 2H), 5.79 (s, 1H), 3.90 (s, 6H), 3.87 (s, 3H), 3.77 (s, 6H). ^13^C NMR (CDCl^3^, 68 MHz): δ 194.8, 162.6, 159.1, 147.5, 145.3, 137.5, 127.6, 126.8, 112.2, 105.6, 91.0, 56.6, 56.2, 55.7. HRMS FAB [M + H] 389.1589.

Procedure C: In a dry reactor, 150 mg (0,76 mmol)of 2-hydroxy-4,6-dimethoxyacetophenone and 139 mg of 4-hydroxy-3,5-dimethoxybenzaldehyde are dissolved in ethanol (4 mL) with 200 µL of piperidine. The reaction mixture is then heated at 150°C for 30 minutes in a Milestone microwave. After cooling at room temperature, the suspension is filtered under reduced pressure leading to 74 mg of the expected chalcone **18** (yield: 27%).

*(E)-3-(4-hydroxy-3,5-dimethoxyphenyl)-1-(2-hydroxy-4,6-dimethoxyphenyl)prop-2-en-1-one* (**18**). ^1^H NMR (CDCl_3_, 270 MHz): δ 14.39 (s, 1H), 7.78 (d, *J* = 15.4 Hz, 1H), 7.71 (d, *J* = 15.4 Hz, 1H), 6.86 (s, 2H), 6.11 (d, *J* = 2.4 Hz, 1H), 5.96 (d, *J* = 2.4 Hz, 1H), 5.81 (s, 1H), 3,95 (s, 6H), 3,91 (s, 3H), 3.84 (s, 3H). ^13^C RMN (CDCl_3_, 68 MHz): δ 192.6, 168.6, 166.3, 162.6, 147.5, 143.2, 137.4, 127.7, 125.7, 106.5, 105.7, 94.1, 91.5, 56.5, 55.9, 55.7.

Demethylation procedure: Chalcone **13** or **15** in solution in 3 mL of dry DCM is treated with 3 eq of AlCl_3_ for three days at room temperature. The reaction mixture is then hydrolyzed with 6 mL of 0.5M aqueous HCl solution and extracted with DCM (3 x 10 mL). The combined organic extracts are dried over sodium sulfate and evaporated under reduced pressure. The phenolic chalcone is purified through flash chromatography.

*(E)-3-(4-hydroxy-3,5-dimethoxyphenyl)-1-(2-hydroxy-4-methoxyphenyl)prop-2-en-1-one* (**12**). The above procedure is applied to 200 mg of chalcone **13**. The conversion rate is estimated at 75% after column chromatography with cyclohexane—EtOAc (8:2) as the mobile phase, 56 mg of **12** as a yellow powder are obtained (brsm yield: 39%). ^1^H NMR (CDCl_3_, 270 MHz): δ 13.54 (s, 1H), 7.84 (dd, *J* = 7.8 Hz, 1.6 Hz, 1H), 7.81 (d, *J* = 15.4 Hz, 1H), 7.42 (s, *J* = 15.3 Hz, 1H), 6.89 (s, 2H), 6.49 (dd, *J* = 8.9 Hz, 2.4 Hz, 1H), 6.48 (d, *J* = 1.5 Hz, 1H), 5.87 (s, 1H), 3.97 (s, 6H), 3.87 (s, 3H). ^13^C RMN (CDCl_3_, 68 MHz): δ 191.9, 166.9, 166.3, 147.5, 145.2, 137.9, 131.3, 126.5, 118.2, 114.3, 107.9, 105.9, 101.2, 56.6, 55.7.

*(E)-1-(2-hydroxy-4-methoxyphenyl)-3-(3,4,5-trimethoxyphenyl)prop-2-en-1-one* (**14**). The above procedure is applied to 45 mg of chalcone **15**. After preparative TLC with cyclohexane—EtOAc (7:3) as the mobile phase, 18 mg of **12** as a yellow powder are obtained. Yield 41%. ^1^H NMR (CDCl_3_, 270 MHz): δ 13.48 (s, 1H), 7.91–7.72 (m, 1H), 7.82 (d, *J* = 15,3 Hz, 1H), 7.46 (d, *J* = 15.3 Hz, 1H), 6.88 (s, 2H), 6.53–6.48 (m, 1H), 6.49–6.48 (m, 1H), 3.95 (s, 6H), 3.92 (s, 3H), 3.88 (s, 3H). HRMS FAB [M + H] 345.1347.

*Ethyl (E)-2-(4-(3-(2,4-dimethoxyphenyl)-3-oxoprop-1-en-1-yl)-2,6-dimethoxyphenoxy)acetate* (**19**). To a solution of chalcone **13** (141 mg, 0.42 mmol) in 7 mL of dry DMF are added 116 mg of potassium carbonate (K_2_CO_3_) (2 eq) and 0.7 mL of ethyle α-bromoacetate (1.5 eq). The reaction mixture is stirred at RT for 24 h and then filtered. After dilution with water, the product is extracted with DCM. The combined organic extracts are dried over anhydrous sodium sulfate. The solvent is removed under reduced pressure. The crude product is purified through silica gel column chromatography using cyclohexane-AcOEt (7:3) as a mobile phase to lead to 92 mg of **19** (Yield: 51%).

*(E)-2-(4-(3-(2,4-dimethoxyphenyl)-3-oxoprop-1-en-1-yl)-2,6-dimethoxyphenoxy)acetic acid* (**20**). Ester **19** (83 mg, 0,21 mmol) is dissolved in tetrahydrofuran (THF) (3 mL) and water (0.6 mL) with 26 mg of lithium hydroxide monohydrate (LiOH.H_2_O) (3 eq). The reaction mixture is stirred at room temperature overnight. After removal of the solvent, the resulting mixture is neutralized and extracted with ethyl acetate (3 × 15 mL). The combined extracts are dried over sodium sulfate, evaporated. The final product is recrystallized in cold isopropyl ether to yield **20** as a yellow solid. (Yield 42%) ^1^H NMR (CDCl_3_, 270 MHz): δ 7.75 (d, *J* = 8.6 Hz, 1H), 7.57 (d, *J* = 15.7 Hz, 1H), 7.40 (d, *J* = 15.7 Hz, 1H), 6.84 (s, 2H), 6.58 (dd, *J* = 8.6, 2.3 Hz, 1H), 6.51 (d, *J* = 2.2 Hz, 1H), 4.65 (d, *J* = 2.6 Hz, 2H), 3.95 (s, 6H), 3.91 (s, 3H), 3.88 (s, 3H). ^13^C NMR (CDCl_3_, 68 MHz): δ 190.5, 170.5, 164.6, 160.7, 152.3, 141.4, 137.9, 133.1, 132.9, 127.9, 122.2, 105.5, 105.3, 98.9, 56.4, 55.9, 55.7.

### 4.2. Calculated logP

Prediction of absorption was performed with the logP calculated with ACD/Labs software predictor (v11.02, Toronto, Canada).

### 4.3. Animals

The present protocol has been approved by the Pays de la Loire (France) ethics committee (20 April 2012) under the register code CEEA.2012.202 and is conformed to the Guide for the Care and Use of Laboratory Animals published by U.S. National Institutes of Health (NIH Publication No. 85–23, revised 1996). Both wild type (WT) and whole-bod knock out (KO) mice were kept at ambient temperature under 12/12-h light/dark circadian conditions and fed standard rodent chow ad libitum and used between 12 to 16 weeks old.

### 4.4. Vascular Reactivity

Female ERα WT or KO C57Bl/6 mice were ovariectomized at the age of 14 ± 2 weeks. After 7 days, the thoracic aortas were isolated and dissected in a physiological saline solution (PSS) (NaCl 130 mM, NaHCO_3_ 14.9 mM, KCl 3.7 mM, KH_2_PO_4_ 1.2 mM, MgSO_4_.7H_2_O 1.2 mM, CaCl_2_.H_2_O 1.6 mM, glucose 11 mM, pH = 7.40) and mounted on a wire myograph filled with physiological saline solution (PSS), at 37 °C, under a flow of a gas composed by 20% O_2_, 5% CO_2_ and 75% N_2_. Mechanical activity was recorded isometrically by a force transducer (Danish Myo Technology, Aarhus, Denmark). Since it has been described that the chalcone isoliquiritigenin exerted a vasorelaxant effect for concentrations ranging from 10^−7^ M to 10^−4^ M (2 × 10^−5^ g/L to 2 × 10^−2^ g/L) [15] and that synthetic anthocynidins exerted a vasorelaxant effect for concentrations ranging from 10^−5^ g/L to 10^−2^ g/L [9], synthetic chalcones were tested for a similar concentrations range (10^−5^ g/L to 10^−2^ g/L). Concentration-response curves were constructed by the cumulative addition of the initial solution of the molecules (0.1, 1, 10 g/L) to reach the following final concentrations in g/L: 10^−5^, 10^−4^, 3 × 10^−4^, 10^−3^, 3 × 10^−3^, 10^−2^. Vessels were pre-contracted with U46619, phenylephrine, and serotonin, at 80 % of their maximal response. The presence of functional endothelium was assessed by determining the ability of acetylcholine (10 µM) to induce more than 50 % relaxation of pre-contracted rings. The same protocol was carried out in presence of 100 µM of L-NAME 20 minutes before the addition of the precontracting agents.

### 4.5. Statistical Analysis

The statistical significance of the differences between samples was determined using the Mann-Whitney test. Differences were considered significant for *p* < 0.05. All statistical tests were carried out with GraphPad Prism 5 (GraphPad Software, Inc, San Diego, CA, USA).

## Figures and Tables

**Table 1:**
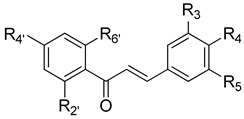
Synthesized chalcones and their related cLogP.

**Compound**	R_2’_	R_4’_	R_6’_	R_3_	R_4_	R_5_	cLogP
**3**	OH	OH	H	OCH_3_	OH	OCH_3_	2.42
**6**	OH	OH	OH	OCH_3_	OH	OCH_3_	1.80
**8**	OH	OH	H	OCH_3_	OCH_3_	OCH_3_	2.75
**10**	OH	OH	OH	OCH_3_	OCH_3_	OCH_3_	2.13
**11**	OCH_3_	OCH_3_	H	H	OH	H	3.20
**12**	OH	OCH_3_	H	OCH_3_	OH	OCH_3_	2.99
**13**	OCH_3_	OCH_3_	H	OCH_3_	OH	OCH_3_	2.84
**14**	OH	OCH_3_	H	OCH_3_	OCH_3_	OCH_3_	3.33
**15**	OCH_3_	OCH_3_	H	OCH_3_	OCH_3_	OCH_3_	3.17
**16**	OCH_3_	OCH_3_	OCH_3_	OCH_3_	OCH_3_	OCH_3_	3.13
**17**	OCH_3_	OCH_3_	OCH_3_	OCH_3_	OH	OCH_3_	2.79
**18**	OH	OCH_3_	OCH_3_	OCH_3_	OH	OCH_3_	2.97
**20**	OCH_3_	OCH_3_	H	OCH_3_	OCH_2_COOH	OCH_3_	2.46

**Table 2:** Summary of the potency and efficacy of chalcones.

Compound	Potency (EC_50_, g/L) WT	Efficacy (E_max_, %)			
		WT	WT with L-NAME	ERα KO	ERα KO with L-NAME
**3**	2.44 × 10^−3^	84.28 ± 3.24 ^a,d^	77.62 ± 4.92	56.23 ± 4.91 ^£^	43.22 ± 6.10 ^$^
**6**	2.12 × 10^−3^	57.10 ± 10.40 ^c,d^	33.92 ± 12.66	60.69 ± 10.75	38.15 ± 10.91
**8**	2.99 × 10^−3^	89.96 ± 4.13 ^b,c^	86.69 ± 3.47	79.73 ± 3.71	69.76 ± 7.99
**10**	4.08 × 10^−3^	63.29 ± 12.79 ^a,b^	44.91 ± 15.07	52.83 ± 12.88	43.33 ± 10.37
**11**	1.93 × 10^−2^	67.35 ± 8.94 ^g^	49.06 ± 9.47	74.81 ± 2.07	36.40 ± 5.81
**12**	NA	30.95 ± 13.23	NA	NA	NA
**13**	5.06 × 10^−2^	80.86 ± 6.11	19.85 ± 7.81 *	81.96 ± 2.46	26.96 ± 6.72
**14**	NA	48.38 ± 7.75	NA	4.64 ± 9.95	NA
**15**	NA	90.59 ± 1.56 ^e,f^	76.07 ± 3.72	79.78 ± 5.51	76.74 ± 5.68
**16**	NA	75.89 ± 5.99 ^f^	NA	74.91 ± 10.15	NA
**17**	NA	72.16 ± 4.59 ^e^	NA	86.88 ± 4.40	NA
**18**	NA	24.34 ± 4.60	NA	NA	NA
**20**	NA	40.77 ± 8.22^g^	NA	NA	NA

NA: Not Available. ^a^ (3 vs. 10, *p* < 0.05), ^b^ (8 vs. 10, *p* < 0.05), ^c^ (6 vs. 8, *p* < 0.05), ^d^ (3 vs. 6, *p* < 0.05), ^e^ (15 vs. 17, *p* < 0.05), ^f^ (15 vs. 16, *p* < 0.05), ^g^ (11 vs. 20, *p* < 0.05), * (WT vs. WT with L-NAME, *p* < 0.05), ^£^ (WT vs. ERαKO, *p* > 0.01), ^$^ (ERαKO vs. ERαKO with L-NAME, *p* < 0.01).

## Abbreviations

ACE	Angiotensin converting enzyme
AlCl_3_	Aluminum chloride
BK_Ca_	Calcium-activated potassium channel-dependent
brsm	Based on recovered starting material
cLogP	Calculated octanol-water partition coefficient
CVDs	Cardiovascular diseases
DCM	Dichloromethane
eNOS	Endothelial nitric oxide synthase
ERα	Estrogen receptor α
ERβ	Estrogen receptor β
EtOAc	Ethyl acetate
EtOH	Ethanol
HCl	Hydrochloric acid
K_2_CO_3_	Potassium carbonate
KO	Knock out
KOH	Potassium hydroxide
L-NAME	Nω-nitro-L-arginine methyl ester
LiOH.H_2_O	Lithium hydroxide monohydrate
MOM	Methoxymethyl
MW	Microwave
NO	Nitric oxide
OCH_3_	Methoxyl
OH	Hydroxyl
PSS	Physiological saline solution
PKG	Protein kinase G
RT	Room temperature
SAR	Structure-activity relationships
sGC	Soluble guanylyl cyclase
SOCl_2_	Thionyl chloride
THF	Tetrahydrofuran
TLC	Thin-layer chromatography
WHO	World health organization
WT	Wilde type
